# Organizational and occupational health issues with working remotely during the pandemic: a scoping review of remote work and health

**DOI:** 10.1093/joccuh/uiae005

**Published:** 2024-01-30

**Authors:** Lynnette-Natalia Lyzwinski

**Affiliations:** Center for Clinical Epidemiology, Lady Davis Institute, McGill Medical School, McGill University, Montreal, Quebec, Canada; Faculty of Humanities, Education, and Social Sciences, University of Luxembourg, Esch-sur-Alzette, Luxembourg

**Keywords:** remote work, telework, COVID-19, well-being, mental health, stress, workplace, productivity, job satisfaction, leadership, work-life balance

## Abstract

Background: Stay-at-home orders during the COVID-19 global pandemic created unprecedented challenges for workers whose work was transferred to the home setting. Little is presently known about the benefits and the challenges associated with global remote work on well-being and mental health, work-life balance, job satisfaction, productivity, home office adaptability, and gender equality.

Methods: A scoping review of PubMed/Medline was undertaken in October 2021 to better understand these broad dimensions associated with remote worker health, well-being, and the home office workspace. The review focused on white-collar workers who undertook remote work during each of the lockdown waves from March 2020 to 2021.

Results: A total of 62 studies were included in the review, which spanned Asia, North America, South America, and Europe. Overall, workers seemed to enjoy remote work, but productivity varied. The main setbacks associated with remote work included feelings of isolation and loneliness, which negatively influenced well-being. Social support from management and contact with colleagues mitigated this. Leadership style also influenced remote worker well-being. Overall, women suffered from lower levels of remote work well-being and productivity, especially if they had children. The home office and its adaptability were integral for successful remote work. Work-life balance was affected in some workers who struggled with heavier workloads or family duties.

Conclusions: To promote well-being and successful remote work, isolation and loneliness should be reduced through greater contact with colleagues and managers. Managers should promote family friendly policies that may support work-life balance and reduce gender inequities in remote work.

## Key points

**What is already known about the topic**
 

- Remote work increased during the pandemic as a result of isolation measures and new working-from-home policies.
- Remote work introduced new challenges and benefits.
- Research on the workplace environment and health has shown that work can impact health and well-being.

**What this study adds**
 

- This research presents the unique challenges and benefits that were identified with working remotely.
- This review addresses health and well-being issues that are specific to remote work, beyond simply exploring the physical work office.

**How might this study affect research and policy?**
 

- The review makes recommendations that could influence organizational working relationships and working-from-home policies.
- These include recommendations for promoting psychological well-being during remote work such as reducing isolation and loneliness.
- It also includes recommendations on organizational leadership and management approaches.
- It makes recommendations for the home office space.

## 1. Background

During the unprecedented COVID-19 pandemic, the mental health and well-being of adults around the globe was impacted.^1^ A systematic review found high rates of depression, anxiety, and stress symptoms in China, Denmark, Sweden, Spain, Italy, Turkey, Iran, and Nepal. Although the prevalence of mental health issues varied by country and assessment method, the prevalence of depressive symptoms was high and ranged from 14% to 48% and 6% to 50% for anxiety symptoms, assessed using validated scales.^1^ Workplace mental health is especially relevant, given the sudden changes in workforce dynamics that the pandemic had brought on. Job loss and financial insecurity were just some stressors that employees faced during the pandemic.^2^^,^^3^ The sudden transition to working from home during lockdown and stay-at-home hygienic public health orders brought on unprecedented stressors for workers including creating a home-office space, work-life balance challenges, adjusting to telework or online work, and lack of clarity surrounding roles.^4^^-^^6^ According to research in Luxembourg, 1 in 3 employees suffered from a decline in their mental health.^7^ In addition to this, perceptions of fear, safety, and losing a loved one during COVID were raised as major issues that working adults faced when trying to adjust to the “new normal.”^8^ It is especially timely to better understand how employers around the world worked towards promoting well-being, buffering against stress and mental health problems in remote workers during times of uncertainty.

Additionally, more research is needed to better understand the benefits and barriers of remote work for well-being and how they vary by gender. Women have been disproportionately affected by the pandemic and they report lower levels of well-being and mental health compared with men,^9^^,^^10^ although this was the case even before the pandemic. They are also affected by inequitable job loss relative to men; 54% of women lost their jobs during the pandemic when they represented only 39% of the working population.^11^ There is also evidence that some women were exposed to increased levels of domestic violence during the pandemic, resulting in new occupational hazards when working from home.^12^ These new work inequities and occupational hazards in the home-office have never been previously studied.

Finally, little is known about the presumed interplay between remote working life, productivity, job satisfaction, work-life balance, and well-being. Table 1. summarizes the various definitions of these constructs, as previously used in the literature. Given that for many people work has moved into the home sphere, the boundary between work and personal life may have become less clear. It is important to understand how remote workers maintained a work-life balance during the pandemic because it is integral for well-being.^13^ There is meta-analytic evidence that stress and mental well-being are associated with job satisfaction,^14^ but it is currently unclear how remote work has impacted global job satisfaction. Additionally, health-related sick leave affects productivity and the economy.^15^ It is presently unclear how productivity was affected during remote working life, and whether inequities exist between men and women, different countries, and diverse white-collar jobs.

**Table 1:** Definitions of essential remote work dimensions.

**Element**	**Definition**
**Work-life balance**	Work-life balance is defined as harmony between one’s personal or private life (eg, leisure, socializing, and family) and one’s working life. When there is imbalance, work may take over one’s time allocated to pleasure and family^18^^,^^19^
**Job satisfaction**	Job satisfaction can be conceptualized as the satisfaction and pleasure with one’s work including finding meaning and purpose in work and with the working built and social environment.^20–22^
**Productivity**	Productivity in the workplace is defined as the output that a person or whole company produces within a period of time during work, sometimes described as the rate at which goods and services are produced.^23^^,^^24^
**Well-being**	Well-being is a state of happiness and living well. Broadly defined by the Centers for Disease Control (CDC), it encompasses positive sentiments and general fulfilment, and spans physical, emotional, social, psychological well-being, and overall life satisfaction.^25^^,^^26^
**Workplace well-being**	Broad concept that includes occupational health and safety and positive perceptions of the workplace.^27^^,^^28^
**Flourishing**	Concept from positive psychology, focused on living one's best life even with limitations (eg, living with an illness or disability).^29^

As remote work has become almost ubiquitous in non–service-related jobs during the COVID-19 pandemic, it is likely to remain to some extent in the future.^16^ Murphy^16^ theorizes that should remote working remain, it will bring its challenges for managers and create gender inequity for women, but employees may find a general preference for this form of work arrangement overall. Kniffin et al^17^ had previously undertaken a scoping review of studies that had been published prior to the pandemic to inform theoretical areas of research including how the pandemic might shape virtual teams, leadership and management, and well-being. A comprehensive updated review is needed that will explore changes in work dynamics, not only in 2020 but also throughout 2021, in order to better understand relationships between remote work, work-life balance, productivity, job satisfaction, and the role leaderships plays. It is also useful for future pandemic preparedness. Gaining a clearer understanding may assist with establishing remote workplace policies that support remote workplace well-being as well as satisfaction with remote work and productivity. Furthermore, interventions may be developed to promote the well-being and success of remote workers by targeting barriers associated with remote work and facilitators. Finally, by better evaluating differences between men and women, future interventions and policies may be better tailored to reduce inequities and promote diverse remote worker well-being.

### 1.1. Aims and objectives

The broad objectives of this review were to summarize the evidence surrounding white-collar employee well-being and remote work. The rationale for focusing on white-collar rather than blue-collar workers is because white-collar jobs could be performed from the “home office” setting during stay-at-home remote work orders when compared with more technical, hands-on jobs, which could only be performed on site by the physical workforce.

The aims of this review were to:

1. Identify the factors affecting employee well-being or acting as buffers against stress while working from home. Determine whether these factors vary by gender and occupation.
2. Better understand how remote work affects work-life balance, job productivity, job satisfaction, and the role of management.
3. Identify the role of leadership and management to foster employee well-being and work under these circumstances.

## 2. Methods

A scoping review of PubMed (Medline) and Google Scholar was undertaken in October 2021 to identify relevant studies to our research questions. The search terms included word variations for “well-being” and “stress” and “remote work” and “work-life balance” and “productivity” and “job satisfaction.” The search strings were combined into 1 large search string that is most relevant to our research questions (Table 2). Keywords included medical subject headings (MESH) terms, free text, word variations, and truncation. A medical librarian was consulted with the search strategy. Manual hand searches of references were also undertaken. The PubMed/Medline search strategy example is given in Table 2.

**Table 2:** PubMed/ Medline search strategy.

((“Personal Satisfaction”[Mesh] OR well being[tiab] OR wellbeing[tiab] OR life satisfaction[tiab] OR personal satisfaction[tiab] OR wellness[tiab] OR “Burnout, Professional”[Mesh] OR fatigue[tiab] OR tired^*^[tiab] OR depress^*^[tiab] OR anxiety[tiab] OR “Mental Health”[Mesh] OR “Stress, Psychological”[Mesh] OR stress[tiab] OR mental health[tiab]) OR job satisfaction OR productivity AND (“Teleworking”[Mesh] OR work from home[tiab] OR working from home[tiab] OR “Telecommuting” OR telecommute[tiab] OR Work[Mesh:NoExp] OR Workplace[Mesh:NoExp] OR Employee[tiab] OR workplace^*^[tiab] OR worksite^*^[tiab] OR “work place”[tiab] OR “work places”[tiab] OR “work site”[tiab] OR “work sites”[tiab] OR “work setting”[tiab] OR “work settings”[tiab] OR “work environment”[tiab] OR “work location”[tiab] OR “work locations”[tiab] OR job[ti] OR work^*^[ti])) AND (coronavirus[tiab] OR “covid 19” OR “2019 ncov”[tiab] OR “2019 ncov”[tiab] OR corona virus disease 2019[tiab] OR corona virus[tiab] OR coronavirus disease 2019[tiab] OR coronavirus[ti] OR coronaviruses[ti] OR COVID[tiab] OR covid19 OR covid-19[tiab] OR nCov 2019[tiab] OR nCov[tiab] OR new corona virus[tiab] OR new coronavirus[tiab] OR new coronaviruses[all] OR novel corona virus[tiab] OR novel coronavirus[tiab] OR novel coronaviruses[all] OR SARS Coronavirus 2[all] OR SARS-COV-2[tiab] OR Severe Acute Respiratory Syndrome Coronavirus 2[tiab] OR “COVID-19”[nm] OR “novel CoV” OR “Coronavirus Infections”[Mesh] OR “Coronavirus”[Mesh] OR “Betacoronavirus”[Mesh] OR “CoV 2” OR “Severe Acute Respiratory Syndrome Coronavirus 2”[nm])

### 2.1. Inclusion and exclusion criteria

This review broadly included all studies in white-collar workers over 18 years of age. Studies must have been undertaken during the COVID-19 epidemic, while employees worked remotely from home. Studies must have been published in the English language or in one of the languages that the co-authors were fluent in such as French, German, Polish, or Italian. Intervention studies were excluded. Studies were excluded if they did not assess well-being, stress, or workplace satisfaction. Studies on pain were included if they measured 1 or more work-related indicators or mental health (stress or well-being as well). Studies were also excluded if they were not undertaken in remote workers, and before COVID-19. Studies that assessed well-being longitudinally including right before COVID-19 and throughout the pandemic were included. Studies were excluded if they primarily evaluated return to work in the actual workplace after lockdown and stay-at-home remote work measures were lifted. Studies were screened against the inclusion and exclusion criteria at the abstract and title, followed by full-text stages.

### 2.2. Data extraction

Data on study characteristics and participant demographics as well as health outcomes and work-related measures were summarized in a tabular format (Table 3).

**Table 3:** Study characteristics.

**Study**	**Design**	**Location**	**Gender**	**Age**	**Employment type**	**Measures**	**Outcomes**
Aczel et al (2021)^19^	Cross-sectional, repeat *n* = 751	N/A	M = 48% F = 50.5%	N/A	Researchers	Productivity Surveys on efficiency of research work, ideal time spent working from home Feasibility of working from home	More efficient *n* = 29% Similarly efficient *n* = 41% Less efficient *n* = 29% Future desire to work from home *n* = 66%
Afonson et al (2021)^20^	Cross-sectional *n* = 143	Portugal	M = 56% F = 44%	Mean 49 (SD = 8.8)	Variable From office workers to medical professionals	Pittsburgh Sleep Quality Index Hospital Anxiety and Depression Scale Perceived job satisfaction + Productivity (self-report Likert Scale)	Poor sleep *n* = 74% Anxiety and depression *n* = 29% Negative relationship between sleep quality and job satisfaction b = −0.17 + Men b = −0.54 Productivity: men b = −0.92 Job satisfaction moderately associated with productivity b = 0.36
Awada et al (2021)^23^	Cross-sectional Repeat from April to June Qualtrics panel *n* = 998	USA	F = 56% M = 32% 11% did not disclose	Mean 40.9 (SD = 13)	Variable Business and office = 29% Engineering 25% Education 22%	Perceived productivity and time spent working at a desk station compared with pre-pandemic times (Likert scale surveys)	Communication with co-workers and productivity *R* = 0.46 (*P* value <.01) Mean increase in work time at a work station =1.5 h/d Productivity higher in workers with 1 specific work station, desk, chair, and no distractions when compared with counterparts (*P* value <.05) Higher mean productivity scores in women (2.94) vs 2.78 in men (*P* value = .05)
Barone et al (2021)^24^	Cross-sectional repeat longitudinal	USA *n* = 112	F = 69% M = 31%	Mean = 45.2(SD = 12.3)	Desk workers Managerial = 64% Admin. = 25% Sales = 5%	Sleep (global sleep quality) Sedentary behavior Emotional well-being Work health Productivity Job satisfaction	No significant changes in sedentary behavior Well-being reduction −6.9 points (SD = 16) (*P* value <.01) Productivity scale reduction of −0.4 points (SD = 1.2) (*P* value <.01) Satisfaction with supervisor = −07 (2.6) and job satisfaction = −0.6 (1.0) (*P* values <.05) Sleep quality improved by 0.7 points (*P* value <.05)
Beno et al (2021)^27^	Cross-sectional	Austria *n* = 154	Cubicle workers M = 44% e-workers M = 13% Cubicle workers F = 33% e-workers F = 9%	Range 20-29 = 16% 30-44 = 38% 45-59 = 24%	Cubicle and e-workers	General working-from-home questions on productivity	Main barriers to productivity: caring for children during work (28%), general routine not productive (26%), less work to do (21%) Desire to work from home always in the future = 22% Often = 35% Sometimes = 27% 15% = never
Bulinska et al (2021)^28^	Cross-sectional	Poland *n* = 220	M = 77.3%	N/A	IT employees Specialists 62% Managers 15% Directors 10%	Job satisfaction	Significant positive association between employee relations and job satisfaction B = 0.367 (*F* = 27.1; *P* value <.01) Mediated by trust in co-workers
Davidsen et al (2021)^32^	Cross-sectional Survey from April to May 2020	Faroe Islands *n* = 1328	F = 77% M = 23%	Mean = 34 Range = 19-71	Public sector(75%) Teaching, health services, child care, social work 19% private sector	Warwick Edinburgh Mental Wellbeing Scale (WEMWBS) Job satisfaction Work-family life (COVID-19 surveys on these domains)	Mean WEMWBS score = 50.7 Challenges with working from home = 37.2% Workers with children had higher stress and lower perceptions related to their work abilities than their counterparts (*P* value <.05) Those who reported job satisfaction were 24% more likely to have a higher well-being score >44 than their counterparts (*P* value <.05) Restrictions impact family life F = 44.5% vs 30% M Social life F = 70% vs 16% M Work F = 70% vs 15% M
Galanti et al (2021)^36^	Cross-sectional	Italy *n* = 209	M = 71.3% F = 28.7%	Mean = 49.8 (SD = 9.4)	N/A	Perceived social isolation Job autonomy Self-isolation Perceived work from home productivity Utrecht Work Engagement Scale Five-point Likert scales applied	Negative relationship between isolation and remote work productivity, $\beta$ = −0.29 (*P* value <.01) Negative relationship between work-family conflict and productivity, $\beta$ = −0.29 (*P* value <.01) Positive relationship with self-leadership and productivity, $\beta$ = 0.17 (*P* value <.01) Positive relationship between work-family conflict and isolation with stress ($\beta$ = 0.31; 0.48) (*P* values <.01)
Garo Albarca et al^37^	Cross-sectional	Costa Rica *n* = 317	M = 81% F = 19%	65% ages 18-29	Software developers	General survey on work	Trust and communication are predictors of the success of virtual teams
George et al (2021)^38^	Cross-sectional	USA *n* = 278	F = 45% M = 55%	Range 22-74 Mean = 39	N/A	General interviews on remote work	Increase in job demands = 44% Prefer to continue working from home = 61% (positive experience) More freedom during remote work = 70% 27% did not find that their productivity increased
Hallman et al (2021)^41^	Cross-sectional From wave of a cohort study	Sweden *n* = 37	81.5% F 18.5% M	Mean = 43.4 (SD = 9.9)	Office workers	Sleep Work time/leisure Using a self-report diary	Mean increase in sleep = 34 min/d Loss of work hours =26 min/d *R*^2^ = 0.74 trust +*R*^2^ = 0.33 communication
Hashim et al (2020)^42^	Cross-sectional	Malaysia *n* = 503	M = 58.8% F = 41.2%	Majority in 30-39 range (53.7%); 40-49 range = 26%	Administrative university staff	Online Likert surveys on job satisfaction, working conditions, and productivity	Job satisfaction with remote work = 87% 35.6% did not have availability of work space at home 35.4% did not have ICT support Work productivity/performance = 53% at home 17.7% did not complete logbooks
Holgerson et al (2021)^43^	Cross-sectional	Norway	N/A	N/A	Wide range of jobs	Survey on job tasks during remote work	38% of jobs may be performed remotely at home Most work-from-home friendly jobs: Managerial =65% Professional = 57.4% Technical = 42.7% Clerical = 63% Sales = 26.7% Agriculture = 17% Trades =12% Mechanical = 7%
Ipsen et al (2021)^6^	Cross-sectional March to May 2020	29 European countries N = 5748 Denmark = 23.3% Germany 23.1% Italy 15.3% Sweden 14.5%	F = 59.2% M = 40.8%	18 to <60 years years of age	Wide range Managerial = 23% Admin = 38.4% Research 20.7% Teaching = 17.9% Sales 5.7%	Survey on working from home experiences	Good experiences with working from home = 55% Cut-off for advantage and disadvantage for remote work mean score >3.5 Advantages: (1) work life balance (2) work efficiency (3) control over work Disadvantages: (1) home office issues (2) insufficient tools (3) uncertainty around work Gender differences: Men adapted better to working from home (Cohen’s *d* = 0.9; *P* value <.01) than women and had fewer office adaptability issues (Cohen’s *d* = 0.76; *P* value <.01)
Krug et al (2021)^47^	Cross-sectional April 11 to May 2, 2020	Germany *n* = 363	F = 68% M = 32%	Mean = 36.3 (SD = 11.0)	Education and science = 16.8% Public service = 15.4% Health and social work = 15.2% Manufacturing and engineering =11% Automotive = 6.6%	Identity Leadership Inventory Social identity with the Exeter identity scales Workplace Loneliness Scale Job satisfaction Kunin Item Scale	Positive association between identity leadership and job satisfaction, B = 041 (*P* value <.01) Negative association between identity leadership and loneliness at work, B = −0.17 (*P* value <.05)
Kruckl et al (2021)^46^	Cross-sectional	Switzerland *n* = 200	N/A	N/A	Health professionals Psychiatric hospital	Depression PHQ-2 Anxiety GAD-2 Stress PHQD	No sign of depression or anxiety Minor stress reported, mean = 2.83 on scale (SD = 2.92) Adaptability of a home office varied by groups and can be implemented in psychiatric hospitals 73% found that Zoom was good for web-based therapy Inadequate IT support = 48%
Lizana et al (2021)^5^	Cross-sectional	Chile *n* = 336	F = 79% M = 21%	Mean = 37.5 (SD = 10.75)	Teachers	Quality of Life QoL (SPF-36) Work hours and work-life balance	Rise in work hours = 78% Work-life balance interruption = 86% Teachers working longer hours were 2 times as likely to report poorer MH outcomes
Lonska et al (2021)^49^	Cross-sectional Sept-Oct 2020	Latvia	F = 79.7% M = 20.3%	Range 25-34 = 20.2% 35-44 = 29.6% 45-54 = 26.8%	N/A	Work-life balance survey	Women and those with children affected Those with children mean household chores score = 3.69 vs 3.29 Women more likely to report increase in household workload, mean = 3.60 vs 3.3 in men (unchanged) (*P* values <.05)
Magnavita et al (2021)^50^	Cross-sectional	Italy *n* = 910	36.6% = M 40.4% = F	Mean = 45.9(SD = 11.3)	Trade and service sector workers	Workaholism: Bergman Work Addiction Scale Stress: Siegrist Effort Reward Balance Mental health: Goldberg Depression and Anxiety Scale Intrusive leadership-toxic leadership scale	Intrusive leadership associated with stress, B = 0.26 (*P* value <.01) + increased work hours/workaholism, B = 0.44 (*P* value <.01) Workaholism moderates the relationship
McDowell et al (2021)^51^	Cross-sectional	USA *n* = 2309	F = 66% M = 34%	Range 18-74	N/A	Mental health: Beck Depression and Anxiety Scale Loneliness Scale Short Warwick Edinburgh Wellbeing Scale	No statistically significant changes in mental health in remote workers No significant differences in loneliness between remote vs non–remote workers
Miyake et al (2021)^*^ (preprint)^53^	Cross-sectional	Japan *n* = 4052	F = 42% M = 58%	Range 20-39 = 20% 40-49 = 28% 50-65 = 52%	Desk workers	Loneliness and job stress (job content) questionnaires	Remote work and loneliness OR (adjusted) = 1.60 ( *P* value <.05; 95% CI exclude 1) Low co-worker support vs high OR (adjusted) = 4.06 (95% CI exclude 1; *P* value <.01) Low supervisor support OR = 2.49 (95% CIs exclude 1; *P* value <.01) Job stress from co-workers = 46% + supervisors = 49%
Moretti et al (2020)^54^	Cross-sectional	Italy *n* = 50	M = 43.1% F = 56.9%	Mean = 46.6(SD = 11.2)	Mobile workers	Utrecht Work Engagement Scale Brief Pain Inventory Fear Avoidance Beliefs Questionnaire	Productivity declined by 30% Stress reduced by 39% Satisfaction comparable to former working conditions = 51% Neck pain intensified = 50%
Sandoval Reyes et al (2021)^58^	Cross-sectional April-May, 2020	Latin America *n* = 1285	F = 69.5% M = 34.1%	N/A	Education = 44% Service sector = 18%	Folkman & Lazarus Work Stress Questionnaire Quantitative Workload Inventory Job satisfaction	Remote work significant predictor of stress: B = 0.269 (*P* value <.01) + negative relationship with work-life B = −0.225 (*P* value <.01) + work satisfaction B = −0.190 (*P* value <.01) Productivity positively related to remote work, B = 0.120 (*P* value <.01)
Savolainen et al (2021)^59^	Longitudinal	Finland *n* = 1308	F = 45.2% M = 54.8%	Mean 45 (SD = 11.4)	Variable	Spielberger State Trait Anxiety Scale Loneliness Scale	Technostress (B = 0.24; *P* value <.01) + loneliness (B = 1.21; *P* value <.01) + stress (B = 0.74; *P* value <.01); associated with poorer MH outcomes (anxiety)
Senturk et al (2021)^61^	Cross-sectional	Turkey *n* = 459	M = 55.3% F = 44.7%	Mean = 35.6 (SD = 6.84)	Software developers = 21.8% Pharmaceutical industry = 17.2% Sales and marketing = 16.1% Bank employees = 14.6% Public office = 6.3% Engineers = 5.2%	Depression, Anxiety, and Stress Scale Jenkins Sleep Scale	Stress = 19.6% Significant differences between men and women Increased workload/hours/chores (*P* values <.05) Female predictor of stress B= 0.155 (*P* value <.01) + loneliness (B = −0.095; *P* value <.05)
Shimura et al (2021)^64^	Longitudinal changes 2019-2020	Japan *n* = 3123	M = 56.7% F = 43.3%	Mean = 37.3(SD = 10.9)	Office workers Tertiary industry	Brief Job Stress Questionnaire Pittsburgh Sleep Quality Index Work Limitations Questionnaire	Decrease in stress compared with period prior to remote work (*F* = 5.42; *P* value <.01) Decrease in work productivity: those who worked remotely 5 d/wk were 1.42 times as likely to be less productive (presenteeism reduced) (*P* value <.05)
Spagnoli et al (2020)^66^	Cross-sectional	Italy *n* = 339	M = 46.6% F = 53.4%	Mean = 48.4(SD = 9.7)	University employees	Toxic Leadership Scale Technostress Creator Scale Dutch Work Addiction Scale	High authoritarian leadership associated with technostress and workaholism vs low authoritarian leadership Significant differences in technostress scores between men and women (women higher) (*F* = 4.5; *P* value <.05)
Staniscuaski et al (2021)^67^	Cross-sectional	Brazil *n* = 3345	F = 68.4% M = 31.6%	N/A	Academics	Survey on work	Productivity negatively affected = 69% Significant differences between men and women in meeting deadlines, X = 21.7 (*P* value <.01) (women less likely) And parents, X = 55.3 (*P* value <.01) compared with nonparents Men without kids met deadlines
Toscano et al (2020)^68^	Cross-sectional	Italy *n* = 265	M = 38% F = 62%	Range 35-36 = 42.3% 36-45 = 21%	Private sector = 59% 40% = public tertiary organizations	General online health and work survey Social isolation (Golden Scale) Stress Scale Remote Job Satisfaction	Both stress (B = −0.17) and social isolation (B = −0.38) have a significant negative relationship with job productivity (p-values <0.01). In addition to this, both stress and isolation have a significant positive relationship B = 0.59 p-value <0.01 Work satisfaction and isolation negative relationship, B = −0.18 (*P* value <.01)
Tusl et al (2021)^69^	Cross-sectional	Germany and Switzerland *n* = 2118	M = 55% F = 45%	Mean = 46.5 (SD = 11.28)	Variable	General work questionnaire Warwick Edinburgh Mental Wellbeing Scale	Work-life worsened = 30% Improved = 10% Work time unchanged 51% Increased in 38% Those who worked from home OR = 2.77 for improved work life vs nonremote Lower well-being OR = 0.61 in workers who had their work life impacted by COVID vs counterparts
Vander Feltz Cornelis et al (2020)^71^	Cross-sectional	UK *n* = 1055	Mean = 45.29SD = 30)	F = 73% M = 26%	University employees	Perceived Stress Questionnaire Mental health-PHQ-9; GAD-7 Presenteeism + Absenteeism-iPCQ	COVID stress = 66.2% Resilience in 33.8% Vulnerability in staff with children OR = 2.23 (95% CIs exclude 1), isolation OR = 1.97 (95% CIs exclude 1); female OR = 1.62 (95% CIs exclude 1)
Xiao et al (2021)^74^	Cross-sectional	China *n* = 988	Mean = 40.9 (SD = 13.1)	F = 56.5% M = 43.5%	Business and office work = 29% Engineering and architecture = 24.6% Computer science and mathematics = 8.2%	General questionnaires on occupational and home office environments General physical and mental well-being (Likert scale)	Communication with co-workers positively associated with improved mental wellbeing, *r* = 0.27 (*P* value <.01) Distractions while working negatively correlated with well-being, *r* = −0.30 (*P* value <.01)
Xiong et al (2021)^75^	Content analysis Cross-sectional	USA Online Twitter *n* = 28 579 posts	30-39 = 37% >40 years = 37% 19-29 = 16%	M = 57.9% F = 42.1%	N/A	VANDER measured sentiment on Twitter/X posts + inference models to extract data on participant characteristics	Women significantly more likely to have +ve attitudes on remote work (*P* values <.01) And higher income earners
Zhang et al (2021)^4^ ^**^	Content analysis	Internet Twitter/X *n* = 1000 tweets	N/A	N/A	Variable	Analysis of themes	Main themes: work-life balance, cybersecurity, effective leadership and teamwork
Almdhawi et al (2021)^21^	Cross-sectional *n* = 299	Jordan	M = 67.9% F = 32.1%	Mean = 46.9(SD = 9.43)	Professors	Health Survey SF-12 Depression and Anxiety Scale-DASS, Neck Disability Index, IPAQ	Increase in work amount = 65% Stress = 17% Overall, good adjustment to working from home Significant relationship between stress (B = −0.48; *P* value <.01) and lower quality of life scores Higher quality of life scores in those satisfied with remote work and teaching (B = 2.81, 3.94; *P* values <.01) Neck Disability Index also negatively related to HRQOL (B = −0.56; *P* value <.01)
Barriga et al (2021)^25^	Cross-sectional	Ecuador *n* = 1044	M = 45% F = 55%	Millennials = 60%	71% = operational positions 28% = supervisors	Work Family Conflict and Burnout Scales	No significant effect of remote work on work-life balance
Chapman et al (20 21)^30^	Prospective cohort	Australia *n* = 163 Academics	F = 72% M = 28%	Range 25-34 = 34% 35-44 = 29% 45-54 = 17%	Academics (*n* = 131)/scientists and nonscientists (admin.)	General survey on work and health	Mental health /well-being negatively affected during remote work = 40% Productivity increased for some groups and tasks: manuscript submissions = increased by 40%. Most often worked in kitchen (42%) and shared home offices (22%) Wore pyjamas during work (46%; scientists) Productivity lower in those with children (63% reduction vs 32% without children) (*P* value <.01)
Castillo et al (2021)^33^	Cross-sectional	Spain *n* = 462 (teleworkers)	M = 39% M = 61%	Mean = 44.6	Variable	General health questionnaire	Remote working associated with lower well-being Women more affected than men
Estrada Munoz (2021)^34^	Cross-sectional	Chile *n* = 3006	F = 71.7% M = 27.4%	N/A	Teachers	Technostress questionnaire	Techno anxiety = 11% Techno fatigue = 7.2% Technostress = 6.2%
Gabr et al (2021)^35^	Cross-sectional	Egypt *n* = 142	M = 52.8% F = 47.2%	Mean = 36.3 (SD = 6.4)	University staff	Technostress questionnaire Blood cortisol	Good Wi-Fi = 78% Technostress higher in teachers without technical support, good Wi-Fi connection, women, not a good home office environment (*P* values <.01) Significant relationship between blood cortisol and technostress (*P* value <.01)
Ghisleri et al (2021)^39^	Cross-sectional	Italy *n* = 211	F = 74% M = 26%	Mean = 53.2(SD = 6.6)	Administrative and technical health care staff	Work–family conflict Cognitive Demands Recovery	Significant positive relationship between ICT stress and work–family conflict (*r* = 0.26; *P* value <.01) Higher work–family conflict in those caring for children (mean score 2.08 vs 1.71 in those without children; *P* values <.05)
Izdebski et al (2021)^44^	Cross-sectional	Poland *n* = 3000	M = 50% F = 50%	Mean = 45 (SD = 16.2)	Variable	Level of Mental Symptoms Index (LMHSI)	Remote work negatively associated with mental health + gender (females reported higher MH symptom scores (62.9 vs 57.6; *P* value <.05) Significant differences in well-being scores and loneliness scores between remote and non–remote workers (*P* values <.01)
Kotera et al (2021)^45^	Cross-sectional	UK *n* = 126	F = 84% M = 16%	Mean = 47.4 (SD = 14)	Psychotherapists	Maslach Burnout Inventory Self-Compassion Scale Work-Life Balance Checklist Telepressure (Likert survey)	Telepressure and emotional exhaustion +ve correlated (*r* = 0.29; *P* value <.01) Self-compassion and telepressure negative relationship (*r* = −0.29; *P* value <.01)
Krukowski et al (2021)^48^	Cross-sectional	USA *n* = 284	Mean = 45.8 (SD = 16.3)	F = 67% M = 33%	Academics	General health and work survey	Women submitted fewer manuscripts (*P* value <.05) Work hours reduced in those with children when compared with counterparts (*P* value <.01)
Meyer et al (2021)^52^	Longitudinal	Germany *n* = 2900	F = 69% M = 31%	Mean = 41.9 (SD = 11.3)	Public admin. = 22.9% Health and social services = 20.5% Universities = 10.7%	Copenhagen Burnout Inventory Perceived social support	Working from home linked with burnout in women with children without access to daycare Women more exhausted than men(b = −0.19; *P* value <.05)
Niu et al (2021)^55^	Cross-sectional	Japan *n* = 5214	M = 77% F = 23%	Mean = 44.2	Office teleworkers teleworkers	Telework survey Work–family conflict scale Kessler Psychological Distress Scale Physical symptoms questionnaire	50% of remote workers satisfied High stress prevalence 30-day prevalence (13% = no stress)
Sharma et al (2021)^63^	Cross-sectional	India *n* = 538	F = 100%	Mean = 32.7 (SD = 7.8)	N/A	General questionnaire created on mental health and work	Mental health declined = 27% (moderate-severe) Increase in household chores = 34% Neck or back pain = 45%
Schade et al (2021)^60^	Cross-sectional	Germany	F = 56% M = 44%	N/A	N/A	Work-related basic needs scale Personal organizational resources Utrecht Work Engagement Questionnaire	Competence associated with productivity Working at home associated with lower relatedness to co-workers (*P* value <.01)
Seva et al (2021)^62^	Cross-sectional	Philippines *n* = 352	F = 62% M = 38%	Median = 35.2	Education = 37% Public admin. = 11.3% IT = 9% Nonmanagerial = 51%	Copenhagen stress questionnaire subscale Nordic musculoskeletal questionnaire	Negative relationship between stress and productivity (B = −0.13; *P* value <.05) Negative relationship between suitability of workstation (B = −0.24) and ergonomic suitability (B = −0.18) and musculoskeletal strain (*P* values <.05)
Weitzer et al (2021)^72^	Cross-sectional	Austria *n* = 686	F = 55% M = 45%	Range <30 = 19.6% 30-39 = 16.8% 40-49 = 26.7% 50-59 = 24%	Variable	General survey on lifestyle, quality of life, productivity	Remote workers 2.07 times more likely to report improved quality of life relative to non–remote workers (95% CI: 1.09-3.91) Less productive when working from home, OR = 1.48 compared with non–remote workers (95% CI include 1) Lower quality of life in mothers of younger children
Wood et al (2021)^73^	Repeat two 4-week diaries Longitudinal	UK *n* = 784	F = 74% first wave 77% second wave	Under 30 = 10% Over 50 = 30-33% waves-2 Phase 2 Mean age 44.56 SD = 11.36 Phase 1 mean age 43.69 SD = 11.10	University staff	Well-being Anxiety, contentment Mental well-being: Warwick Edinburgh scale Job demands Social support Work to nonwork conflict Loneliness ICT constraints	Well-being declined Isolation/loneliness increased
Yoshimoto et al (2021)^76^	Cross-sectional	Japan *n* = 1941	M = 70.5% F = 29.5	Median = 43	Variable	General pain, working style, stress questionnaires	Pain increased = 15% Stress increased = 47% OR telework = 2.27 (95% CI exclude 1) for pain OR for pain from stress = 2.216 (95% CI exclude 1)
Andel et al (2021)^22^	Longitudinal 8-week diary	USA *n* = 265	F = 44.5% M = 55.5%	Mean = 40.8 (SD = 4.6)	Professionals = 20.4% Manufacturing = 11.3% Retail = 12.8% Technical = 18.9%	Telecommuting behavior Self-compassion Depression	Telecommuting frequency associated with work loneliness (*r* = 0.12, *y* = 0.42; *P* value <.01) Mindfulness moderated this relationship For work loneliness (*y* = −0.6) and depression (y = −0.13) (*P* values <.05)
Bennett et al (2021)^26^	Cross-sectional	USA *n* = 55	M = 58% F = 42%	Mean = 33.6 (SD = 9.0)	Legal services, banking, finance, health care, education, IT	Qualitative thematic analysis With a general health survey on working from home	Negative relationship between work group belongingess and Zoom fatigue (*y* = −0.21; *P* value <.01)
Chan et al (2021)^29^	Cross-sectional	USA 151 elementary schools	M = 19% F = 81%	N/A	Teachers	Emotional exhaustion: Maslach Burnout Inventory Job demands Teaching efficacy and school connectedness	Belonging (school connectedness (*r* = −0.27; *P* value <.05) + autonomy linked with job satisfaction (*r* = −0.72; *P* value <.01) Satisfied with job less than sometimes = 23%
Conroy et al (2021)^31^	Cross-sectional	USA *n* = 834	F = 78.7%	Mean = 43 (SD = 12.9)	Psychologists =21% Physicians = 18% Nurses = 10%	General health survey	Mood worsened = 57% Went to bed later = 64% Woke up later = 74% Significant differences between remote and non–remote workers (*P* value <.01)
Guler et al (2021)^40^	Cross-sectional	USA *n* = 194	M = 51% F = 49%	Mean = 33.9(SD = 7.6)	Engineers = 35.6% Academics = 11.9% Analysts =12.4% Software specialists = 9.3% Bankers = 6.2%	General questionnaire on work and health Nordic Musculoskeletal Questionnaire	Productivity increased (1-3 h = 28%) and back pain (*P* value <.01) Reduced working hours = 15% Chair comfort = uncomfortable 12.4% Unsure 22% Shared working room = 12% always, often 18%
Okawara et al (2021)^56^	Cross-sectional	Japan *n* = 5760	M = 58% F = 42%	Range 20-65	Office and non–office workers	Work Functioning Impairment Scale	Participants who did not have adequate lighting in their home offices more likely to have work impairment OR = 2.02 (95% CI exclude 1)
Shockley et al (2021)^65^	Longitudinal 7 weeks follow-up	USA *n* = 334 couples	N/A	Mean F = 34.8 (SD = 5.7) M = 36.3 (SD = 6.6)	N/A	General questionnaires on health, family, work performance	Most of the housework undertaken by women = 36.6% Women had the lowest well-being scores when they did all of the family work while working remotely
Abdel et al (2021)^18^	Prospective cohort 7-day diary	Germany *n* = 178	N/A	N/A	Variable	General questionnaires on job and home demands, leisure, and work performance	Home demands positive relationship with exhaustion (B = 0.46; *P* value <.01) and work demands (B = 0.11; *P* value <.01) with emotional exhaustion Leisure negative relationship with exhaustion (B = −0.11; *P* value <.01) Negative relationship between job performance and emotional exhaustion (B = −0.12; *P* value <.01)
Usman et al (2021)^55^	Two-wave time lagged Cohort	Pakistan *n* = 236	F = 73.7% M = 26.3%	<25 = 24.2% 25-33 = 58% >33 = 16.9%	Health care	Participative Leadership Scale Work thriving	Participative leadership associated with work thriving (B = 0.57; *P* value <.01)
Salazar et al^57^	Cross-sectional	Spain *n* = 677	M = 50% F = 50%	Mean = 48.7(SD = 10.5)	University workers	Depression and anxiety DASS-21 Coping strategies Cope-28 Brief Illness Perception Questionnaire	Social support inversely related to depression (rho = −0.169), anxiety (rho = −0.115), and stress (rho = −0.108)

DASS, ; F, ; GAD-2, ; HRQOL, ; ICT, ; IPAW, ; iPCQ, ; IT, ; M, ; MH, ; N/A, ; OR, odds ratio; PHQ-2, ; PHQD, ; SPF-36, VANDER, WEMWBS.

## 3. Results

**Figure 1:**
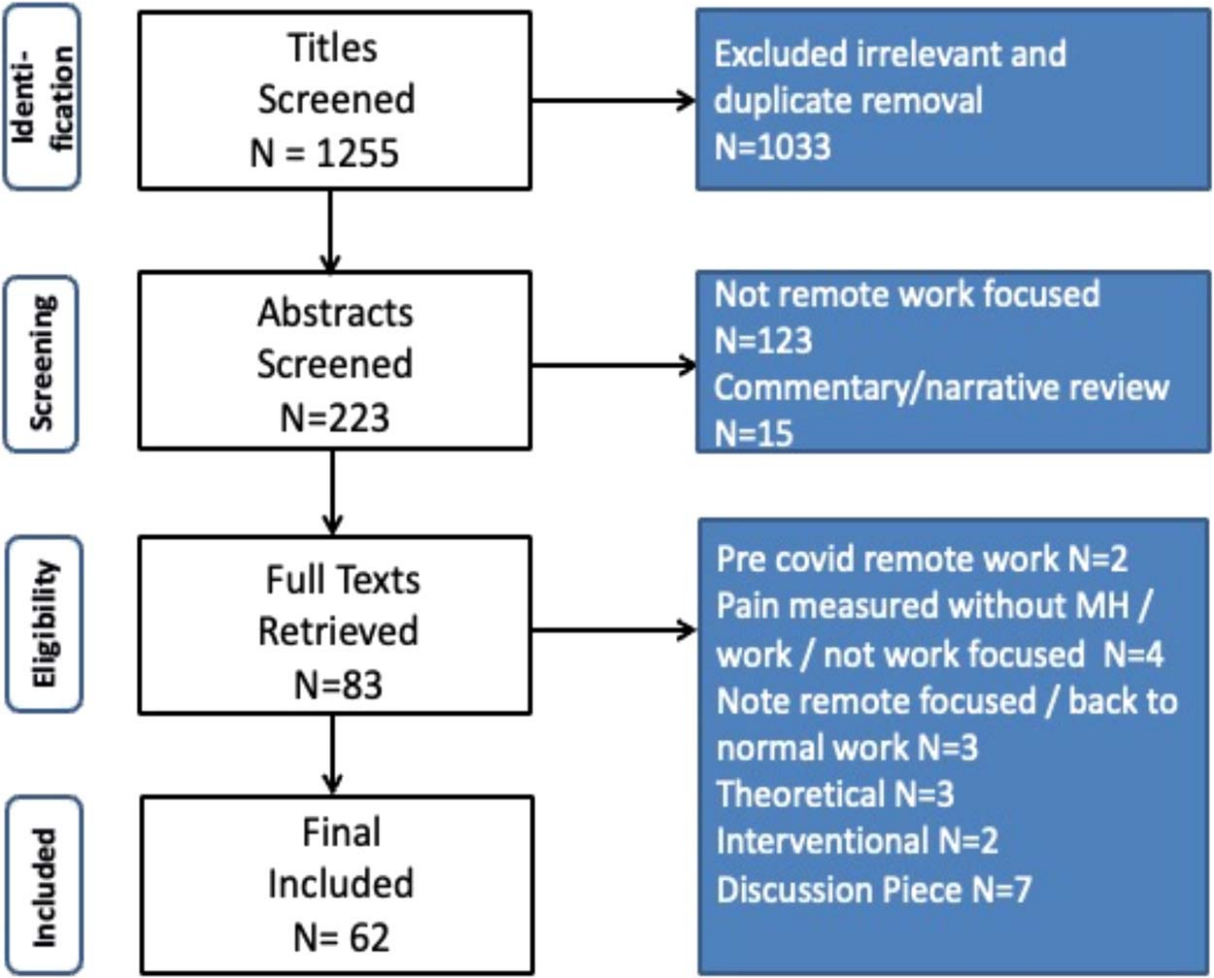
Preferred Reporting Items for Systematic Reviews and Meta-Analyses (PRISMA) flow diagram of the search.

After title and abstract screening, followed by full-text retrieval, 62 studies were included in this review and are summarized in Table 3.^4–6^^,^^18–76^ The studies spanned Europe, North America, South America, Asia, and the Asia Pacific Region. The employment categories were diverse, but most often included public sector workers such as managers, office workers, administrators, researchers, and teachers. The age of workers varied, but most often covered middle-aged working adults. The search and screening process at each stage based on the key inclusion and exclusion criteria is summarized with the Preferred Reporting Items for Systematic Reviews and Meta-Analyses (PRISMA) flowchart in Figure 1.^77^

### 3.1. Remote work and well-being

#### 3.1.1. Effects of remote work on well-being and stress

The impact of remote work on well-being, stress, and mental health varied across the studies.^19^^,^^21^^,^^30^^,^^31^^,^^33^^,^^44^^,^^45^^,^^55^^,^^64^^,^^72^^,^^73^^,^^76^ Whereas some studies reported benefits of working from home on well-being, others found that well-being declined. For example, a study in Australia found that mental health was negatively affected in 40% of remote workers.^30^ Similar findings were reported in Europe, including in studies in Spain^33^ and in Poland.^44^ A study of university staff found reduced overall well-being and increased stress^73^ in the United Kingdom. Higher levels of tele pressure from working online were associated with emotional exhaustion in employees.^45^ Likewise, studies in Japan found a high 30-day prevalence of stress in teleworkers of 87%,^55^ and increases in stress by 47%, when cross-sectionally evaluating perceived changes in stress during the pandemic.^76^ Nevertheless, some studies also found that working from home was associated with improved overall well-being.^19^^,^^21^^,^^64^^,^^72^ For instance, 1 study in Japan found that remote work reduced overall stress in employees and promoted mental well-being,^64^ which contrasts with the aforementioned studies in Japan. Similarly, research in Austria found that employees were twice as likely to report improved quality of life relative to their non–remote-working counterparts.^72^ Studies in professors found that many enjoyed working from home, across different countries.^19^^,^^21^ Specifically, a study in academics found that 66% of them supported future remote work for their well-being.^19^ Thus, working from home was not uniformly associated with poorer well-being and varied according to other contextual factors including employment type.

#### 3.1.2. Social isolation as a barrier to remote workplace well-being

Working from home during the pandemic was associated with isolation and loneliness, which were found to influence well-being and stress during remote work. Several studies found that social isolation played a role in employee well-being or mental health when working from home.^22^^,^^36^^,^^44^^,^^47^^,^^53^^,^^59^^,^^61^^,^^68^^,^^69^^,^^71^^,^^73^^,^^74^^,^^78^ For instance, to demonstrate relative differences between remote and non–remote workers, a study in university employees who reported that they were isolated were 78% more likely to be vulnerable to poor well-being and higher stress when compared with their counterparts who did not feel isolated.^71^ Approximately one-quarter of university staff experienced depression or anxiety.^71^ In contrast, a large cross-sectional study in the United States during early lockdown in April 2020 did not find that working from home was associated with higher levels of loneliness.^51^ Technostress was identified as a predictor of poor mental health outcomes in workers in Finland.^59^ Nonetheless, the trends point towards vulnerability to feelings of isolation and loneliness when working from home, which influence remote workplace well-being, stress, and mental health.

#### 3.1.3. Employee social support as a buffer for remote workplace well-being

Co-workers and employers play a role in providing employees with social support to buffer against the negative effects of isolation and loneliness when working from home and to promote well-being.^26^^,^^47^^,^^53^^,^^57^^,^^69^^,^^79^^,^^80^ Regular social support was found to be integral for well-being,^81^ mitigating the effects of loneliness and isolation associated with remote work. Social connectivity was identified as a main factor for buffering against stress in 1 study in Austria,^79^ whereas a study in Spain found that social support was negatively associated with depression.^57^ Levels of contact with co-workers influenced perceptions of loneliness.^53^ A study found that remote workers were 1.6 times more likely to experience feelings of isolation when compared with non–remote workers and that having co-worker support mitigated this.^53^ They also found that remote workers with little support from co-workers were 4 times more likely to report feelings of isolation when compared with those with high levels of support.^53^ Zoom fatigue was found to be inversely related to group belongingness in the workplace,^26^ highlighting the need to promote interconnectedness during telework or online remote work.

Management played a role in fostering social support and reducing feelings of isolation in the workplace. Organizational support, which is support from the organization in which one works, was found to be a significant determinant of remote workplace well-being^80^ and fostered a sense of identify and belonging in the workplace.^47^ Workers with low levels of managerial support were 2.5 times more likely to report loneliness when compared with those who reported high levels of support.^53^ Thus, it appears that not only co-worker support, but also managerial support is important for fostering a remote workplace environment that promotes well-being and reduces feelings of loneliness.

In addition to direct support from the workplace and colleagues, having personal support in one’s private life was also identified as being important for buffering against the negative effects of working from home. For example, the negative impact of working from home on personal life was mitigated by having a partner when compared with single individuals who were younger.^69^ Therefore, remote workers should ideally have support on a spectrum ranging from colleague and co-worker social support and regular contact, social support from middle management, and peer as well as family social support when working remotely to promote well-being.

#### 3.1.4. Gender differences in remote workplace well-being

Women reported higher levels of stress and lower levels of well-being than men in several studies.^32^^,^^33^^,^^34^^,^^59^^,^^61^^,^^65^^,^^71^ One study noted that women’s well-being scores were contingent on the amount of housework, with women experiencing lowest levels of well-being when they undertook the majority of housework while they were teleworking.^65^ Similar findings on unequal role play were noted in another study, whereby women took on greater roles in family activities.^75^

Childcare or having children was also associated with poorer overall mental health or well-being.^32^^,^^52^^,^^59^^,^^61^^,^^71^^,^^72^^,^^74^ For example, women were 1.6 times as likely to be vulnerable to the stressors from COVID, and those with children were 2 times as likely, relative to their male counterparts and those without children.^71^ Interestingly, a study estimated that remote work may lead to inequality as it benefits men and those in the higher income brackets, and could lead to higher income for these groups.^82^ Another study recommended equitable childcare accessibility for women when working from home,^83^ supporting research indicating that women experience burnout especially when they work remotely and cannot access childcare.^52^ Productivity was also reported to be significantly lower in women in Australia if they had children when compared with those without children (reduction of 63% vs 32%).^30^ Thus, there is a need to better understand the unique differences between men and women and to offer tailored supportive interventions in order to reduce inequalities.

### 3.2. Work-life balance

In addition to social support, isolation, and the role of organizational support, other challenges associated with remote work life that have been identified include work-life balance and work–family conflict issues. Work-life balance associated with remote working life was measured using subjective self-report, and the findings were mixed across the studies.^6^^,^^32^^,^^36^^,^^39^^,^^58^^,^^69^^,^^84–86^ A large study in Germany and Switzerland found that those who worked from home were 2.77 times more likely to report improved work-life balance compared with non–remote workers. The exception was when the lives of employees were directly affected by COVID-19.^69^ Likewise, a large study in Europe found that improved work-life balance was a benefit of working from home.^6^ There was variability between countries, however, when considering remote work-life balance perceptions during the pandemic. For example, a study in the Faroe Islands found that 37% struggled with working from home.^32^ Likewise, a study in Latin America found that remote work during the pandemic reduced work-life balance.^58^

It appears that work-life balance also varied between different professions. A large study in Europe found that part-time teleworkers had a good work-life balance, but that this balance was compromised in individuals who worked full time and were mobile (needed to move for work).^84^ A study found that work-life balance was not affected if employees had regular employment, instead of under precarious employment conditions.^85^ Work-life balance also varied across different professions. A study in teachers found that 86% felt that telework caused difficulties with maintaining work-life balance, primarily due to increased work hours.^5^ Increased work hours in tandem with work-life imbalance were predictors of poor quality of life on the mental health part of the survey.^5^ Higher workloads and monitoring of work from home led to greater conflicts in the home-work sphere.^86^ Higher levels of stress and isolation were also negatively associated with work-life balance.^36^^,^^39^ Thus, ways to reduce excessive work hours and stress are needed and stress coping skills may also be useful to improve work-life balance in remote work.

### 3.3. Productivity and work time

Productivity varied across studies and was measured mostly through self-report,^6^^,^^18^^,^^20^^,^^23^^,^^24^^,^^27^^,^^36^^,^^37^^,^^42^^,^^54^^,^^64^^,^^67^^,^^68^^,^^72^ which may limit conclusions to be drawn from these results as such self-reports are open to bias.^87^ Whereas some studies found increases in productivity during remote work,^6^ others found reductions in productivity.^54^^,^^64^^,^^67^^,^^72^ A large study in Europe found that productivity increased when working remotely from home as well as greater control over work.^6^ A study in Italy found that 39% of employees reported reduced productivity during remote work,^54^ and a study in Brazil similarly found that 69% of workers felt that their productivity had declined.^67^ Research in Austria found that remote workers were 1.48 times more likely to be unproductive when compared with their non–remote-working counterparts.^72^ Research in Japan found that productivity or presenteeism (being present during virtual work by completing online log books) declined when workers worked remotely on a full-time basis.^64^ A study involving 704 academics found that productivity decreased in 50% of the sample, but approximately 25% of the academics found that they were more productive when working from home during the pandemic.^19^ However, they found that 70% were willing to work from home and could build on their work efficiency from this experience, which made them better prepared to undertake remote work in the future.^19^ Academics reported that they could work better in the traditional workplace when communicating with colleagues and undertaking necessary data collection, but they could focus more on data analysis and on the literature when working from home.^19^ Thus, it seems that productivity changes were not uniform when working from home, but also depended on the tasks, and that this transition suited some individuals and professions more than others. It also seems that adjusting and building habits associated with remote work is important for supporting productivity.

Factors that affected productivity throughout the studies included caring for children^27^ or work–family conflicts,^36^ lack of regular office space (including a reserved desk and chair),^6^^,^^23^^,^^42^ distractions,^23^ lack of habit formation for working from home^27^ or self-leadership,^36^ isolation^36^ or emotional stress,^18^^,^^68^ communication with co-workers and trust,^37^ satisfaction with remote work^20^ and with the relationship with the supervisor,^24^ and IT support.^42^ Productivity was also affected by higher levels of communication with co-workers and less isolation in tandem with overall good health.^23^ Thus, it seems that in order to stabilize or even increase productivity, employers should aim to improve communication with employees and their co-workers, reduce isolation, assist with an adequate home work-station setup, and promote a work-life balance. Productivity may also vary by income or type of employment. Older individuals and those in a higher income bracket were more likely to report being productive during remote work,^23^ indicating that there may be an inequality gap. A study in Norway further found that 38% of jobs can be performed remotely.^43^ This highlights that inequalities in remote work accessibility and capability to perform productively exist.^43^

#### 3.3.1. Working time

In addition to productivity, 3 studies explored changes in work time during remote work. A large study in the United States found that working time increased by 1.5 extra hours of work each day when working from home,^23^ whereas another study in the United States found that 28% of workers increased their work hours by 1-3 hours during remote work.^40^ By contrast, in a Swedish study, individuals who worked from home slept for 34 minutes longer, which took away time from work.^41^ Thus, it is unclear whether work time increased or decreased for workers around the globe, given the conflicting findings. However, it should be noted that longer work hours do not necessarily linearly equate with increased productivity, and shorter work-weeks have been suggested in previous research for increasing productivity.^88^

#### 3.3.2. Gender differences in productivity

Gender differences in productivity were reported across some studies, but the results were variable. Men were more likely to report poor levels of productivity,^20^ and stress affected productivity more in men than in women in 1 study in Latin America.^58^ Likewise, in another study, women in the United States were found to report higher levels of productivity when working from home during remote online work or “telework.”^23^ An analysis of Twitter/X posts in the United States found that women were more likely to post positive things about remote working life.^75^ By contrast, a study in Latvia found that women had more chores than men and that they were more likely to suffer from work-life imbalances.^49^ Similarly, a study in Turkey found that women had increased workloads, work hours, and chores, and were more likely to report stress than men.^61^ Women in the age group 18-44 were particularly negatively affected by remote work relative to men in 1 study.^61^ Women were also found to have higher technostress scores than men in Italy^66^ and in Egypt.^35^ A large study in Europe found that men adapted to working from home better than women and had fewer office adaptability issues.^6^ A study in Brazil in academics found that women were less productive than men when submitting manuscripts during the pandemic, especially if they had children.^67^ Similarly, a study in academics in the United States found that they submitted significantly fewer manuscripts when working remotely from home.^48^ Thus, it appears that productivity was lower in females in the academic profession, but more research is needed to better understand whether this was a consistent trend. Likewise, it appears that there may be gender inequalities in home-office adaptability as well as balancing chores at home. Understanding reasons for differences in productivity and well-being, especially levels of support, role sharing in housework, and accessibility to childcare, is important for reducing inequalities in the homework sphere.

### 3.4. Job satisfaction

Job satisfaction was measured by self-report questionnaires^6^^,^^20^^,^^24^^,^^28^^,^^29^^,^^32^^,^^38^^,^^42^^,^^47^^,^^54^^,^^58^^,^^68^^,^^85^ and differed between the studies. A study across 29 European countries found that 55% of workers had an overall good experience when working from home.^6^ A study in Latin America, however, found that job satisfaction declined during remote work.^58^ By contrast, job satisfaction in Italian employees working from home remained the same for 50% of participants^54^ when compared with their former satisfaction in the traditional workplace. The exception was that workers who had neck or back pain were less likely to be satisfied with remote work.^54^ A study in Malaysian university employees found that 87% were satisfied with working from home, although their reported productivity was only 54%, due to gaps in the availability of telework technical support and equipment.^42^ A study in the United States found that 61% of workers enjoyed working at home and that 70% felt that they had more freedom.^38^

Different factors across the studies that may affect remote job satisfaction include the type of contract (level of security), overall well-being and sleep, isolation, and work relations.^20^^,^^28^^,^^29^^,^^32^^,^^47^^,^^58^^,^^68^^,^^85^ A study found that employees had higher levels of job satisfaction when working from home only when they had a strict regular employment contract and not working under uncertain, precarious, or irregular employment conditions.^85^ Another study found that 74% of teleworkers reported poor sleep quality, which was inversely related to job satisfaction.^20^ Maintaining positive relations at work might also be integral for job satisfaction during the pandemic and that trust was a mediator between positive relations and job satisfaction.^28^ Autonomy,^29^ social identity in the workplace, and connectivity with co-workers were also found to be key determinants of remote work job satisfaction,^47^ whereas social isolation was associated with lower job satisfaction^29^ and overall productivity^68^ as well as stress.^68^ Satisfaction with the quality of supervision was also related to satisfaction^24^ as well as overall employee relations, including with co-workers and trust as a fundamental element^28^ and identity leadership.^47^ There was also a positive relationship between well-being and overall remote job satisfaction^32^ and a negative relationship with stress and satisfaction.^58^ Thus, finding ways to improve relationships at work, improve sleep, and reduce stress may be beneficial for job satisfaction during telework.

### 3.5. Home office adjustment

The home office as a physical space was identified as being an integral element of remote work in various domains including well-being and productivity. This includes a consideration of the desk, the table, the room, lighting, or technical aspects such as Wi-Fi.^6^^,^^23^^,^^30^^,^^35^^,^^40^^,^^42^^,^^56^^,^^59^^,^^74^ Research in Japan found that home office lighting was integral for workers and that those who had inadequate lighting were 2 times as likely to suffer impairments in their work.^56^ Wi-Fi connectivity was not ideal for 22% of university staff in Egypt and was associated with technostress.^35^ Research in Australian academics found that most did not have access to a home office and worked primarily from their kitchens (42%), whereas others shared an office space (22%).^30^ Interestingly, in the United States, 12% reported sharing office space when working remotely across diverse professions.^40^ Workers who had an adequate room to work in reported higher productivity compared with those who did not have acceptable home office adaptabaility.^23^^,^^42^ Owning desk space was associated with working longer during remote work.^23^ Adequate workspace, workstation, lack of distractions, and regular work hours were associated with higher well-being in remote workers in one study.^74^ A large study from 29 European countries found that the main setbacks and barriers associated with working from home during the pandemic were inadequate home office space/adaptability as well as insufficient tools available to employees.^6^ Thus, the home office space and its various dimensions are integral for supporting successful remote work and well-being.

There is also some evidence that employees reported greater physical pain from ergonomic stressors when working from home. One study recommended an ergonomic work station for home office work and well-being during the pandemic,^89^ supporting previous research that found that 50% of employees reported greater neck pain when working from home.^54^ Overall work-related health indicators declined in remote workers in one study.^24^ Another study found a negative relationship between home desk and chair ergonomic and overall suitability and musculoskeletal strain.^62^ Thus, employers should make efforts to reduce ergonomic stressors by providing their employees with suitable adjustable chairs and workstations. In addition to this, a study that investigated home office adaptability in health care workers found that not all could adapt equally to the home office, especially those who required seeing patients, technical support, or when hand on work at the actual work setting was necessary to complete the tasks.^46^ Thus, the home office should ideally be created to maximize working opportunities, but even with adjustments, some professions are not suited for home-based work.

### 3.6. Leadership style

In addition to managerial support, which was described previously, the actual leadership style played a role in shaping remote worker well-being.^37^^,^^47^^,^^50^^,^^61^^,^^66^^,^^70^^,^^90^ Participative leadership was an important determinant of employee health, whereby employees were empowered to be active decision makers in the workplace.^70^ Another study found that identity leadership, through greater group cohesion and sense of collective belonging in the workplace, was positively related to work satisfaction and negatively related to loneliness during remote work.^47^ Additionally, leadership style was found to moderate the effect on well-being of working from home. For example, intrusive leadership was found to be negatively associated with mental health and well-being in workers (increased depression, anxiety, and reduced happiness).^50^ In other words, pushing workers to be workaholics through excessive pressure was found to be detrimental to well-being.^50^

Communication and trust in leaders were found to be integral during remote work, especially doing virtual teams.^37^ Low levels of perceived control in the work setting and higher workloads were associated with lower levels of mental health and well-being.^61^ A study in Italian university workers and administrators found that highly authoritarian leadership was detrimental to work-life balance, as it promoted workaholism and led to increased levels of technostress (stress associated with using technology).^66^ Likewise, intrusive or toxic leadership was found to be associated with higher levels of stress in a study in Italy in trade and service sector workers.^50^ Thus, this form of leadership demonstrates negative effects in diverse employees. Another study suggested that trust in leaders, effective communication, empowerment, and social cohesion are all integral for successful virtual teams.^37^ A study suggested that responsible management and strategic leadership are essential for workplace well-being and overall performance when working from home.^90^ Work uncertainties were identified as key setbacks associated with remote work in a large study across Europe,^6^ highlighting the need for clear direction, role identity, and organizational leadership. Interestingly, not only leadership by top management, but also self-leadership in the form of self-direction and personal initiative were important for productivity during remote work.^36^ Thus, employees also seem to have a role in managing and organizing themselves in their new home-work office sphere.

## 4. Discussion

The aims of this study were to review the relationship between remote work and well-being, including its determinants, as well as its relationship with work-life balance, productivity, job satisfaction, and home office adaptability. This review also aimed to better understand the role of management and leadership in promoting well-being and ideal remote working conditions.

The dimensions associated with remote work life in employees who worked at home during the COVID-19 pandemic have been reviewed. This is important given that it explored various dimensions of remote workplace health and well-being in depth on a global scale. It is novel as it focused on several key areas that are interconnected, including remote workplace well-being and its determinants, work-life balance, productivity, the roles of management and leadership, job satisfaction, and home office adaptability. The review also compared differences in gender across each dimension, highlighting inequities in remote worker well-being and aspects of work at home. The review was not limited to early stages of lockdown in March 2020 but included research on remote work throughout 2021 (second and third lockdown waves), demonstrating some longer-term effects of remote work rather than focusing on only the immediate adjustment over the short term. Although this review found variability across studies, including between countries and different employment sectors, several emergent themes were identified. Their implications for the workplace and well-being will be discussed.

First, many employees enjoyed working at home overall. Nevertheless, social isolation and loneliness were identified as barriers in many studies. This review found that remote workplace loneliness has an impact on well-being and stress. Finding ways to engage workers with their co-workers and with their managers is one solution that may ease isolation, given that several studies found that co-worker and employee support are integral. It is no surprise to find that social support is a prerequisite for employee well-being when working remotely from home. Previous Whitehall II studies on workplace health found that social support was integral for workplace well-being, supporting the findings in this review.^91^ A previous review similarly recommended that employers should provide social networking opportunities for employees when working from home.^81^ Some commercially available apps have been recently developed that aim to provide a platform for employees to connect and feel less isolated, such as SquadPal,^92^ which is a social remote work messaging app for co-workers. More research is needed to develop evidence-based resources for employees. There is a need for future public health interventions to be tailored to the current needs of remote workers, by targeting the determinants of remote work isolation and loneliness.

Second, work-life balance may be positively or negatively impacted by remote work, which was also found to influence well-being and stress. This is contingent upon regulation of work hours, and supervisory support. A review suggested that companies will need to find ways to manage the boundaries between work and private life for future remote workers.^81^ Productivity is both positively and negatively affected by remote work, depending on the contextual living situation. Factors that need to be considered include work office adaptability and accessibility to IT support, which were identified as determinants of successful remote work from home. Employers should provide employees with desks, ergonomic chairs, and other necessary work tools that will promote workplace well-being and productivity in the home-work sphere. Similar to our review, previous reviews found that employers should provide employees with sufficient technical support when working from home.^78^^,^^81^ Another review had suggested that companies should have working from home indicators,^81^ and another suggested that employee skills training is needed.^93^ Finally, productivity may be affected by childcaring, hence the need for universal daycare accessibility, not only but especially when people need to work from home.

Third, this review found that leadership plays an important role in remote work. This not only includes leadership style, by being less authoritative or “toxic,” but also includes effective communication and trust building. Toxic leadership was associated with higher stress and was not conducive to well-being but rather to workaholism.^50^^,^^66^ This review found that employees had better overall experiences when they had good relationships with their supervisors as well as co-workers. A review suggested that companies should readjust their structure to be more equitable and less hierarchal, whilst ensuring a fine balance between productivity and taking care of the well-being of employees.^94^ Another review concluded that levels of organizational support are integral for remote workplace well-being.^95^ Indeed, effort-reward balance and level of control and decisional support had been previously identified as being integral to workplace well-being and health in the classic Whitehall studies in workers.^91^ Similar strategies should be applied to remote work, whereby managers should aim to promote effort-reward balance and provide employees with greater control.

Self-leadership and identity also appear to be important, as employees who had a clear direction of what they were doing and had created a routine in their remote work life, had a better experience when working from home. Thus, it appears that “it takes two to tango” when working remotely, as one must have self-discipline in tandem with effective leadership from supervisors. A review also suggested that leaders should clarify roles for employees, to set a clear direction when working from home.^81^

Fourth, attention needs to be paid to special groups, especially women and those with children who may find it challenging to cope with both work and childcare. Nevertheless, this review did find that some women enjoyed working from home more than men. Reasons for differences between studies could be due to factors such as accessibility to childcare. Supporting women through universally accessible childcare may reduce any inequalities^96^ from remote work. Both gender and access to childcare have been identified as some of the social determinants of health by Raphael,^96^ highlighting that these determinants need to be targeted by policymakers in order to ensure that working from home will be equitable. A review also found that women did not have improvements in their health when working from home.^81^ Additionally, more studies on remote work and domestic abuse are needed, to better understand the needs of vulnerable populations who are working from home and to develop policy and public health interventions.

In addition to gender, more research is needed in diverse groups to better understand diverse needs. One study examined the effects of disability on remote work and found that individuals with a disability were more likely to work from home and could benefit from this new adjustment in the workplace setting.^97^ Thus, future research could further explore the dynamics between remote work and disabled workers, to support equity when working from home. Additionally, there is a gap in the remote work literature, whereby differences according to age group, ethnicity, and socio-economic status were not evaluated. Future studies should explore these dimensions to better understand and address health and work inequities. Finally, as many studies used self-report questionnaires assessing constructs of interest, there is a need for greater validation of these measures and more universal scales to measure this emerging work-related and well-being dynamic in the home-work sphere.

### 4.1. Conclusion

In summary, this review found that remote workplace well-being varies on a continuum, and factors such as employment characteristics, gender, isolation and loneliness, co-worker support, leadership style and support, as well as home office conditions all play a role. Whereas some workers had a positive experience with remote work, others experienced challenges. Remote work may promote well-being if conditions are supportive and when barriers such as isolation, loneliness, and work-life imbalances are reduced. Productivity and job satisfaction may be enhanced through greater support from managers and by offering employees support with their office setup, and by providing women and those with children with adequate accessibility to childcare.

## Funding

None declared.

## Conflicts of interest

No conflict of interest to declare.
